# Editorial Comment: Two-stage Fowler-Stephens orchidopexy in management of undescended testes: Is it time for a change? A UK multi-centre retrospective study

**DOI:** 10.1590/S1677-5538.IBJU.2025.9919

**Published:** 2025-10-20

**Authors:** Luciano A. Favorito

**Affiliations:** 1 Universidade do Estado do Rio de Janeiro Unidade de Pesquisa Urogenital Rio de Janeiro RJ Brasil Unidade de Pesquisa Urogenital - Universidade do Estado do Rio de Janeiro - Uerj, Rio de Janeiro, RJ, Brasil; 2 Hospital Federal da Lagoa Serviço de Urologia Rio de Janeiro RJ Brasil Serviço de Urologia, Hospital Federal da Lagoa, Rio de Janeiro, RJ, Brasil

**Ahmed Mohamed ^1^, Caroline Mary Macdonald ^2^, David Keene ^3^, David Marshall ^4^, Gregory Shepherd ^5^, Karim Awad ^6^, et al.**

^1^ Royal Belfast Hospital for Sick Children, 274 Grosvenor Rd, Belfast, BT12 6BA, United Kingdom; Alder Hey Children's NHS Foundation Trust, E Prescot Rd, Liverpool, L14 5AB, United Kingdom; ^2^ Sheffield Children's NHS Foundation Trust, Clarkson St, Broomhall, Sheffield, S10 2TH, United Kingdom; ^3^ Royal Manchester Children's Hospital, Oxford Rd, Manchester, M13 9WL, United Kingdom; ^4^ Royal Belfast Hospital for Sick Children, 274 Grosvenor Rd, Belfast, BT12 6BA, United Kingdom; ^5^ Nottingham Children's Hospital, Queen's Medical Centre, Derby Rd, Lenton, Nottingham, NG7 2UH, United Kingdom; ^6^ Bristol Royal Hospital for Children, Upper Maudlin St, Bristol, BS2 8BJ, United Kingdom; Ain Shams University Hospitals, Al-Khalifa Al-Ma'mun Street, Cairo, Egypt.

**J Pediatr Surg. 2025 Sep 11;60(12):162665.**

DOI: 10.1016/j.jpedsurg.2025.162665 | ACCESS: 40945709

## COMMENT

Testicular migration is a complex process (1). In cases where the testicles are not palpable, laparoscopic or open surgical exploration is necessary to identify the morphology and anatomical locations of the testes, vas deferens and testicular vessels and thus select the most appropriate surgical technique to avoid testicular damage (2). Most orchidopexies can be performed through inguinal or even scrotal access. Laparoscopy is often used to diagnose non-palpable testes, by assessing testicular position if the testes are atrophic or absent. Laparoscopy allows simultaneous performance of orchidopexy with high success rates. Two-stage Fowler-Stephens surgery is one of the surgical options to treat high abdominal undescended testis (3).

In the present paper of Moahmed and collegues (4) the authors assessed outcomes of staged Fowler-Stephens orchidopexy (FSO) in management of intra-abdominal testes (IAT) and concluded that atrophy rates after staged FSO ranged from 11 % to 37 % across 6 paediatric surgery tertiary centres in the UK. Atrophy rates are higher than expected and a prospective study is warranted to compare FSO with alternative operative techniques such as gubernaculum-sparing and traction orchidopexy.

## Data Availability

Not applicable
